# Depression, Anxiety and Stress among nursing officers in a dedicated hospital for COVID patients in Sri Lanka: A Single Institute Experience

**DOI:** 10.12669/pjms.38.4.5508

**Published:** 2022

**Authors:** Pathirajage Deepthi Madushan Pathiraja, Wallagoda Samantha Srikanthi, Bernard Deepal Wanniarachchi Jayamanne, Hewage Sanduni DeSilva

**Affiliations:** 1 Pathirajage Deepthi Madushan Pathiraja, MBBS, MD, MSLCOG, MRCOG. Consultant Obstetrician and Gynaecologist, The Ministry of Health, Sri Lanka; 2 Wallagoda Samantha Srikanthi, BSc-Nursing, Nursing Officer, Department of Obstetrics, Colombo East Base Hospital, Sri Lanka; 3 Bernard Deepal Wanniarachchi Jayamanne, MBBS, MSc (Biostatistics), MSc (Biomedical), MIASSL, Faculty of Medicine, University of Kelaniya, Sri Lanka; 4 Hewage Sanduni DeSilva, MBBS.Senior Medical Officer, Department of Obstetrics, Colombo East Base Hospital, Sri Lanka.

**Keywords:** Depression, Anxiety, Stress, Nursing Staff, COVID-19 pandemic, DASS 21

## Abstract

The main objective of this study was to assess the prevalence of stress, anxiety and depression, among nurses working in a tertiary hospital dedicated to the COVID-19 patients in Sri Lanka. A cross-sectional study was carried out among nurses working at Colombo East Base Hospital. The data was collected using a self-administered questionnaire and DASS-21, a set of three self-report scales designed to measure the negative emotional states of depression, anxiety, and stress over three months from October 2020. Data were analysed applying descriptive statistics and inferential statistical methods. There was a total of 131 study participants (response rate 83 %), and most of them were working in general wards (56%), while 42% were in critical care units. The proportion of anxiety and stress is associated with nurses working in critical care units were significantly higher than those in general wards (p<0.001). There were no associations between sex, marital status, having children, experience, qualifications, and medical or psychiatric conditions (p>0.05). The system of reporting mental health issues was unfortunately not in place. Staff felt that reporting stress/burnout or anxiety might seem like a negative attribute. Considering the above factors, one would expect more prevalence than we have seen in this study; therefore, we can infer that if mental health is not prioritised in healthcare institutions, then definitely lack of awareness/openness and under-reporting by staff will result in a long-term systemic problem (Suffering in the name of Resilience).

## INTRODUCTION

Global pandemics have occurred in regular intervals in the past few decades. The health system has faced immense changes during SARS (severe acute respiratory syndrome), MERS (Middle East Respiratory Syndrome), and the H1N1 pandemic. World Health Organization (WHO) declared the COVID-19 a global pandemic in March 2020.^1^

Studies have shown that pandemics exacerbate the stress and anxiety levels among health care staff as they cope with intense physical, emotional, and cognitive demands.^2^,^3^ The psychological well-being of health care members is conditioned by the resources available at the settings and dedication by the governing bodies. To ensure safe, consistent practice, accurate data on current experiences are vital. Especially, those who work as front-line workers are subject to additional stress as they directly engage with infected patients.^4^,^5^ On the other hand, there was a fear of disease being transmitted to their family members, concerns about their children health and safety, fear of being stigmatised and rejected in the community, especially in different cultural backgrounds.

## METHODS

This survey was conducted in Colombo East Base Hospital (CEBH), Colombo, Sri Lanka. All frontline nurses who were directly involved with the care of COVID-19 positive patients were invited to the study. Nurses who already had COVID-19 infection and/ or pregnant women were excluded.

A self-administered questionnaire was developed to recruit the socio-demographic, personal health, and work-related characteristics. Further, Depression Anxiety and Stress Scale (DASS) scale were used to assess the psychological wellbeing of the study participants. The DASS is a 21-item self-report instrument developed initially by Lovibond et al. (1995) to measure the three negative emotional states of depression, anxiety, and stress level.^6^

It has been validated to Sinhala and Tamil versions and is freely available.^7^ Each sub-scale consists of seven items and is rated using a four-point scale, ranging from zero to three. (e.g., 0=“ did not apply to me at all” and three “applied to me very much or most of the time”). The total score was calculated by summing up the scores on each item on the sub-scale. This questionnaire is a public domain and requires no permission for use.^8^

An informed written consent was obtained from the participants. The researcher collected data over a period of three months from October 2020 onwards. Data were coded, entered on a spreadsheet (Microsoft Excel), and analysed by employing descriptive and inferential statistical methods using SPSS software (version 22). The study was conducted under the ethical standards and criteria and approved by the ethics committee at National Institute for Mental Health (NIMH), Colombo. Ref -(168/12/2020)

## RESULTS

All frontline nurses who were directly involved with the care of COVID-19 positive patients were invited to the study, and a total of 131 respondents (response rate 83 %) were recruited. Most nurses were working in general wards (56.5%), while 42%, 2.3% worked in the critical care units and administrative departments, respectively. The majority were females (85.6%), and the mean age was 35.5 years (±8.6). 76% of study participants were married, and 80% lived with their families. Further, 80% of married respondents had children. Nearly 70% (n=92) had less than five years of experience, and 26 (n=34) had bachelor’s degrees in addition to the basic diploma. We noted that 15% had medical comorbidities (diabetes, hypertension, asthma), and 2 % had psychiatric issues such as mild personality and eating disorders and were on regular medications.

Results revealed that 29.9% were depressed, 29.8% were suffering from anxiety, and 19.8% were stressed at various levels (Table-I). The proportion of depressed, with anxiety and stressed with other socio-demographic factors are listed in Table-II.

**Table-I:** Levels of depression, anxiety and stress among the study group.

	Depression (%)	Anxiety (%)	Stress (%)
Normal	103(78.6)	92(70.2)	105(80.2)
Mild	13(9.9)	08(6.1)	13(9.9)
Moderate	10(7.6)	14(10.7)	07(5.3)
Severe	03(2.3)	12(9.2)	05(3.8)
Extremely Severe	02(1.5)	05(3.8)	01(0.8)

**Table-II:** Proportion of depressed, with anxiety and stressed with socio-demographic factors of the sample.

Factor		Depression		Anxiety		Stress	

		Yes	No	Yes	No	Yes	No
Sex	Male	04 (26.7%)	11 (73.3%)	83 (71.6%)	09 (60.0%)	05 (33.3%)	10 (66.7%)
	Female	24 (20.7%)	92 (79.3%)	33 (28.4%)	83 (71.6%)	21 (18.1%)	95 (81.9%)
Marital status	Married	21 (21.0%)	79 (79.0%)	33 (33.0%)	67 (67.0%)	20 (20.0%)	80 (80.0%)
	Other	07 (22.6%)	24 (77.4%)	06 (19.4%)	25 (80.6%)	06 (19.4%)	25 (80.6%)
Have Children	Yes	20 (25.0%)	60 (75.0%)	26 (32.5%)	54 (67.5%)	17 (21.2%)	63 (78.8%)
	No	08 (15.7%)	43 (84.3%)	13 (25.5%)	38 (74.5%)	42 (82.4%)	09 (17.6%)
Living with family	Yes	23 (21.9%)	05 (19.2%)	31 (29.5%)	74 (70.5%)	20 (19.0%)	85 (81.0%)
	No	82 (78.1%)	21 (80.8%)	08 (30.8%)	18 (69.2%)	06 (23.1%)	20 (76.9%)
Experience	< 1 year	05 (38.5%)	08 (61.5%)	05 (38.5%)	08 (61.5%)	05 (38.5%)	08 (61.5%)
	1- 5 years	07 (26.9%)	19 (73.1%)	10 (38.5%)	16 (61.5%)	07 (26.9%)	19 (73.1%)
	> 5 years	16 (17.4%)	76 (82.6%)	24 (26.1%)	68 (73.9%)	14 (15.2%)	78 (84.8%)
Qualifications	Diploma	18 (18.6%)	79 (81.4%)	26 (26.8%)	71 (73.2%)	15 (15.5%)	82 (84.5%)
	Bachelor	10 (29.4%)	24 (70.6%)	13 (38.2%)	21 (61.8%)	11 (32.4%)	23 (67.6%)
Working unit	General	12 (16.2%)	62 (83.8%)	16 (21.6%)	58 (78.4%)	07 (09.5%)	67 (90.5%)
	Critical	16 (29.6%)	38 (70.4%)	22 (40.7%)	32 (59.3%)	19 (35.2%)	35 (64.8%)
	Admin	0 (0.0%)	03 (100.0%)	01 (33.3%)	02 (66.7%)	0 (00.0%)	03 (100.0%)
Medical issues	Yes	08(40.0%)	12 (60.0%)	08 (40.0%)	12 (60.0%)	05 (25.0%)	15 (75.0%)
	No	20 (18.0%)	91 (82.0%)	31 (27.9%)	80 (72.1%)	21 (18.9%)	90 (81.1%)
Psychiatric illnesses	Yes	01 (33.3%)	02 (66.7%)	01 (33.3%)	02 (66.7%)	01 (33.3%)	02 (66.7%)
	No	27 (21.1%)	101 (78.9%)	38 (29.7%)	90 (70.3%)	25 (19.5%)	103 (80.5%)

The mean age of nurses with anxiety group (33.4 years) was found to be significantly lower than those without anxiety (36.4 years) (p-value =0.046). Further, a significant association between the unit that they are working with their anxiety (p-value =0.03) and depression levels (p value= < 0.001). There are no associations with sex, marital status, having children, experience, qualifications, medical or psychiatric issues (p-value > 0.05) (Table-III). There was a significant strong positive correlation among the three domains. The Spearman correlation coefficient was 0.84(p <0.001), 0.85(p <0.001) and 0.87(p <0.001) respectively.

**Table-III:** Association of factors with depression, anxiety and stress status by Chi square test.

Factor	df	Depression		Anxiety		Stress	

		Test statistic	p value	Test statistic	p value	Test statistic	p value
Sex (Male/Female)	1	0.038	0.84	0.385	0.53	1.098	0.29
Marital status (Married/Other)	1	0.000	0.99	1.505	0.22	0.000	0.99
Have children (Yes/No)	1	1.101	0.29	0.435	0.51	0.078	0.78
Living with family(Yes/No)	1	0.000	0.97	0.000	0.99	0.035	0.85
Experience (<1 y, 1-5 y, > 5y)	2	3.603	0.16	2.006	0.37	4.889	0.09
Qualifications (Diploma/Bachelor)	1	1.178	0.28	1.074	0.30	3.515	0.06
Unit (Critical / Other )	1	2.549	0.11	4.589	0.03*	11.220	< 0.01*
Medical issues(Yes/No)	1	3.652	0.06	0.674	0.41	0.104	0.75
Psychiatric diseases^§^ (Yes/No)	1	0.520	0.52	0.000	0.99	0.351	0.49

* Significant associations;

§ Fisher exact test.

## DISCUSSION

Sri Lanka’s healthcare system is governed primarily by the public sector.^9^ During the pandemic, an overwhelming impact on the mental aspect of health care workers became evident, which would impose a considerable effect on their mental wellbeing.^10^,^11^ It was apparent that significant association of depression and anxiety scores with the unit that they are working (p=0.004). Working conditions of the different units, especially the direct contact with infected patients, may have contributed to these findings. In addition, the workload, support available and restrictions to personal lifestyle at various levels may have been different. Similar results were reported in studies conducted in other south Asian countries.^12^,^13^

Considering the unprecedented manner and high risk involved with the pandemic, some degree of stress, anxiety, and even insomnia must be anticipated. However, further clinical assessment is warranted to confirm the presence of depressive disorder in those with high scores. Furthermore, detailed assessments of contributory psychological factors are needed. Comparatively, high reported anxiety and stress levels were observed in nurses who worked in critical care units. A system of reporting mental health issues is unfortunately not in place.^14^ The staff reported stress, burnout, or anxiety at the workplace as a negative attribute or weakness. Further, Stigmatisation regarding psychological issues, lack of awareness about these impacts on quality of life and patient care. The lack of understanding about concepts like burnout may have contributed to the aforementioned findings. A cross-sectional survey among health care workers in Pakistan showed how much they performed their duties diligently during the outbreak; however, they were much concerned and were afraid of infecting their family members.^12^ The authors highlight the importance of continued moral and financial support by the government and other health care organizations to appreciate their services.

Further, it was observed that the nurses belonging to the younger age group experienced a higher degree of anxiety. Less resilience to workplace stressors and limited experience may have contributed to this difference. Similar results were demonstrated by a study among nurses in a tertiary care hospital of India where anxiety and depression levels were more in the younger and less experienced nurses.^13^ A systematic review by Sonja et al. revealed an increased risk of stress-related disorders, fear of the unknown or becoming infected were at the forefront of the mental challenges faced. Being a nurse and being female appeared to confer more significant risk.^15^

This study highlights the importance of psychological awareness and support for the health care workers facing the current pandemic. Considering the issues of underreporting, stigma, and lack of awareness, it is justified to expect a higher prevalence of psychological problems than the findings of this study. Therefore, interventions to address these gaps in services need to be established to improve the psychological wellbeing of the health care workers. These will enhance the quality of life of the service providers and improve the quality of patient care and health services. Change needs to start at the level of policymakers to offer an enhanced variety of supports to nurses and other health care workers who play a critical role during large-scale disease outbreaks.

### Authors’ Contribution:

**PDM:** Conceived, designed, writing & editing of manuscript, and final approval.**WAS:** Conceived, Data collection and final approval of manuscript.**BDJ:** Data analysis and final approval. **SDS:** Data collection, writing of manuscript and final approval of manuscript.
